# Hospital and Institutional News

**Published:** 1914-01-17

**Authors:** 


### HOSPITAL AND INSTITUTIONAL NEWS.

### DEATH OF A LONDON HOSPITAL CHAIRMAN.

The death of Mr. Ernest Arthur Lazarus-Barlow, brother of the medical man alluded to in our article on the "Cancer Cure" controversy elsewhere in this issue, removes an apostle of personal service from the voluntary hospitals. Among the many charities with which he was associated special mention must be made of the French Hospital, to which he was successively treasurer and chairman of the board of management, in which capacity he received President Poincaré when he visited the hospital last year. The Legion of Honour was conferred on him for his services to the institution and to the French colony, any charity in connection with which immediately awakened his interest.

### A PERSONAL NOTE.

We have received the following personal note: "Mr. Ernest A. Lazarus-Barlow, who died suddenly on the 9th inst. at his residence at West Mersea, was always glad to escape thither, when his many activities would permit, to indulge in his favourite pastime of yachting. He succumbed, at the age of fifty-eight, to a second attack of apoplexy, a previous one having been nearly fatal. He was one of the directors of the Comptoir National d'Escompte de Paris, and manager of the London agency of that financial institution. In 1893 he became the hon. treasurer of the French Hospital, and in 1909 was elected by his colleagues as chairman of the committee. He was a man deeply attached to the interests of the hospital and its associated convalescent home at Brighton, and his business instincts and clear vision were a most valuable aid to the development and prosperity of the work carried on. His generosity was also most delicately bestowed on both institutions."

### ILLEGAL RESOLUTIONS BY SOUTH SHIELDS GUARDIANS.

Boards of Guardians often pass illegal resolutions which have to be quietly rescinded subsequently or allowed to remain a dead letter. Two recent examples, however, are of importance because they illustrate how the powers given to the medical officers of Poor-Law institutions by the Orders of the Local Government Board enable those officers to protect the inmates from injustice, and how important it is that those powers should remain undiminished by maintaining the status of the medical officer and his independence as regards the Guardians and the Guardians' other officials. In South Shields the Guardians have adopted a resolution, moved by Mr. H. E. Lincoln, that intoxicating liquors requisitioned by Dr. J. Dudgeon Giles, the medical officer, for the use of his patients be no longer supplied. This resolution is distinctly illegal. The Orders of the Local Government Board give to the medical officer absolute discretion as to the diet and stimulants to be given to any sick inmate, and the master is compelled to supply all diets, extras, and stimulants properly requisitioned by the medical officer. Any refusal to obey the instructions of the medical officer in this matter would place the master in a very serious position. He could be dismissed by the Local Government Board for not carrying out the duties of his office, and, in the event of the patient's death being caused or hastened by his neglect to supply the stimulants ordered, he might have to answer to a more serious charge. The most extraordinary thing that occurred at the meeting was that Mr. J. Gill, the chairman, who explained to one member the legal position of the clerk, was one of those who voted in favour of refusing further requisitions.

### INFIRMARY OFFICERS AND MATERNITY BENEFIT.

The other instance, referred to above, occurred at Bethnal Green. Here the Board of Guardians passed a resolution that no officers shall sign any certificate or other paper to enable the mother of an illegitimate child, born in an institution of the Guardians, to receive maternity benefit unless such mother shall give a written authority to the post office or her approved society to pay the whole of the thirty shillings direct to the Guardians. This resolution, of course, chiefly affects the medical officers, and as far as they are concerned it has no binding force at all, because it is contrary to article 205 (3) of the General Order of July 1847, which makes it the duty of a medical officer to give a certificate "to the pauper on whom he is attending, of the sickness of such pauper or other cause of his attendance when required to do so." Parliament having decided that a mother, whether married or not, if a worker, is entitled to maternity benefit, Guardians have no right to try to penalise the unmarried mother. Fortunately, we may confidently rely upon the medical officers to ignore a resolution which is both illegal and unjust.

### A WOMAN PIONEER OF LUNACY REFORM.

A notable personality in the history of the modern lunacy laws passes away with the death of Mrs. Georgina Weldon in the mid-seventies of her life. The obscurity in which this lady's last years were shrouded was as complete as the publicity connected with her name in the 'seventies and 'eighties, as the celebrated heroine of numerous cases in the Law Courts. In 1878 an attempt was made, at the instigation of her friends and relatives, to spirit her away to a private asylum on the apparent ground that her philanthropic project, teaching orphans, and her separation from her husband implied some mental aberration. She baffled her would-be captors, and as soon as the Married Woman's Property Act of 1882 allowed a woman to sue in the courts without her husband's consent she instituted proceedings against Dr. Forbes Winslow, under whose care she was to have been placed. After having her case debarred from going to a jury by Baron Huddleston, the Divisional Court, in 1884, accorded her a verdict of £1,000 damages against one of the medical men who signed the certificate of lunacy. The trial, which lasted ten days, excited immense public interest, and focussed attention on the horrors permissible under the then Lunacy Laws. Her renewed efforts against Dr. Winslow at length resulted in a verdict of £500 damages against him for assault. A London paper was mulcted of a similar sum in the same year for libel, but the next year she herself was sentenced to six months' imprisonment for libelling M. Rivière, the musician. She emerged from prison, however, under a writ of *habeas corpus*, to gain £10,000 damages against Gounod, the composer of "Faust," for libel, another musical friend with whom she eventually quarrelled. In the same year, 1885, £1,000 damages were awarded her against Sir Henry de Bathe, who, it was stated, had given the order, at her husband's request, on which the medical certificate for her detention as a lunatic was granted. Her interest in the reform of the Lunacy Laws is shown in the lectures she gave and the periodical that she edited on the subject. She even went on the stage to act in a play entitled "Not Alone," the object of which was to expose the evils which the law permitted. She was engaged to sing at the Pavilion—quite in the modern manner. How far she was from being fit for incarceration may be judged from the fact that she conducted her own case in all the actions which she undertook.

### THE NEW HEART INSTITUTION OPENED.

On Monday last Prince Arthur of Connaught opened the new buildings of the National Hospital for Diseases of the Heart, in Westmoreland Street, Marylebone, of which we publish an illustrated description on another page. The hospital's President, Lord Verulam, received Prince Arthur and accompanied him to the out-patient waiting-hall, where the opening ceremony took place. The Prince recalled that this was the second move that the hospital had made since its foundation in 1857 in a small house in Margaret Street. First, in 1874, it moved to 32 Soho Square, and now, forty years later, it is installed for the first time in a specially designed building. In speaking of the electro-cardiograph the Prince remarked that it had been devised by one of the consulting physicians to the hospital, Professor A. D. Waller. Prince Arthur then declared the building open and received purses containing £191 for the institution.

### LONDON CHILDREN AND MOTOR-AMBULANCES.

The London County Council has an ambulance service for conveying crippled children to and from the special schools, and now an experiment is to be made with a motor-ambulance, which is expected to do the work of four horse-vehicles. There are 2,400 children who have every day to be taken to school, and the service costs about £13,000 per annum. There are now fifty-five vehicles in use, but the number will be considerably reduced if the motor-ambulance proves successful. Motor-ambulances are in use by the Metropolitan Asylums Board, which reports that the results warrant their gradual increase.

### TRUANT SCHOOLS AND ALTERNATIVE REFORMS

Dr. M. L. Tyler, of the London Homœopathic Hospital, has drawn attention to the case of an epileptic boy of six, brought to the institution after being struck on the head by the radiator of a motor omnibus. Dr. Tyler explains that this child had been charged before a Clerkenwell magistrate for truancy, and sentenced to three years' attendance at a "truants' school," near Drury Lane. His home is in the north of London, and, it seems, he had to get up at 6 a.m. to wait for a boy aged nearly eleven to pilot him to school, and back again between 7.30 and 8 at night. The pilot liking to perch on the back of carts, his protégé had to cling on to the chain, from which one day he fell, and thus was sent to hospital. This ghastly story—imagine how Dickens would have told it!—leads Dr. Tyler to condemn the system which makes magistrates relegate epileptic children of six to attend schools, with apparently no thought of the distance they may have to travel, and the consequent risk they run and also cause in the London streets. If it is necessary to have truant schools, they should be resident schools, since, as this case shows, opportunities for truancy and something much worse are bound to flourish so long as only daily attendance is demanded. Or, failing that, an ambulance as exists for the special schools, should fetch the children to and fro. We hope that Dr. Tyler will continue his investigations, and inform us of the possibility of reorganising the truant school system by either method.

### ONCE MORE THE BRITISH HOSPITAL FOR MENTAL DISORDERS.

In successive issues we have called attention to the unsatisfactory state of affairs which prevails at this so-called hospital. *Truth* has recently directed its eagle eye, we are glad to know, to the abuses which exist at this institution, and in its issue of the 31st ultimo it sets forth a letter from Messrs. Parker and Thomas, solicitors to the hospital, of 18 Ironmonger Lane, E.C. The investigations made by *Truth* show that an average of only four new out-patients a week enter the hospital waiting-rooms, whilst the attendances are only thirty per week. The cost per attendance is 2s. 10½d., which is unjustifiably great. Since Dr. Hollander retired the medical staff consists of three gentlemen—one a general practitioner practising near the hospital, the second a general practitioner living in Brighton, and a third gentleman residing at an hotel. *Truth* states that the two former attend a few hours one day each week, and the third has not been seen at the hospital for many months. The Editor properly insists that: "A medical man in general practice cannot be expected, at the same time, to be in a position to specialise in such a difficult branch of his profession as the treatment of nervous and mental disorders and brain disease. The 'patient who goes" to this so-called hospital "can get no advice specially applicable to his case that he could not obtain as an out-patient at any of the big general hospitals. Any medical institution which spends over 45 per cent. of its income on salaries and 'collectors' commissions stands self-condemned.'' Dr. Hollander came to the conclusion that the way this institution is managed lays it open to the aspersion that it is not being kept up solely for the benefit of the patients, but for the benefit of those who draw the salaries and commissions from the funds collected.

### STREET COLLECTING AND NEW INSTITUTIONS.

If, as we understand, its main source of revenue is from moneys collected by boxes in the streets we venture to hope the Chief Commissioner may be moved to investigate the matter. He has it in his power to refuse to license habitual collectors throughout London for any so-called institution of an unnecessary and undesirable type. It would appear, however, from a rather scandalous case reported in the London papers on the 10th instant that the police are not enforcing the regulations which were obtained with great difficulty mainly through our action when we led an agitation to abolish street collections in London on behalf of the Hospital Saturday Fund. Public opinion has long been in favour of the abolition of collectors and collecting boxes from the streets. The history of the Hospital Saturday Fund proves that such collections do harm, and not good, to *bona-fide* charities. Hence Sir Edward Henry can render great public service if he will give a little personal attention to street collections and prevent them from taking place in any portion of the Metropolis. Another question arising out of the so-called British Hospital for Mental Disorders is the urgent need in this country for legislation which would empower County Councils to exercise control over new so-called hospitals and charities by instituting a licence without which no building should be allowed to be called a hospital, or to be established at the will and pleasure of any adventurer in any portion of the Metropolis. Strong evidence was given on this point before the House of Commons Committee on the Registration of Nurses in 1905, and we commend it to the immediate and careful study of the London and County Councils and their officers.

### A COAT OF ARMS FOR THE ASYLUMS BOARD.

In order to increase the prestige of the Metropolitan Asylums Board, for that was the specific argument, Mr. Mount Somerby urged the Board at a recent meeting to apply for a coat of arms. The cost, he said, would be £131 10s., and this would be more than returned by the increased prestige of the Board. So many people had failed to hear of the Board that the need for placing some distinctive device at the entrance of its institutions had long been contemplated. The proposal, supported by Canon Sprankling, the vice-chairman, and by Sir Francis Fleming, was unanimously adopted by the Board. We confess that there seems to us a confusion in the argument of Mr. Mount Somerby. We do not believe that the prestige of the Metropolitan Asylums Board would be increased by a grant of arms, and such a consideration in any case has little bearing upon the question of whether the institutions under the Board's control should bear a common device, appearance, or lettering. The cipher M.A.B. has a prestige which no other device connected with the Board could possibly equal; it has, moreover, the sanction of popular usage. If it is desirable, which is another question, to make each institution controlled by the Board to proclaim its connection, let the famous cipher be placed on every building and property. We dare say that there is a good deal to be said for objectifying the reality of the Board in this way, but it would not cost £131 10s. to adopt our suggestion, and we defy anyone to suggest a more impressive device than the famous letters which are used by everyone who has ever heard of the Asylums Board, and are to-day more likely to excite interest and respect than any crest in those who have not.

### THE WESTMINSTER SITE.

Sir J. Wolfe Barry, chairman of the house committee of Westminster Hospital, desires it to be known that the site of the institution in Broad Sanctuary is still open for purchase, the governors' negotiations with the Canadian Government with reference to the erection of a Dominion building in London having fallen through. The space is really one which ought not to be built on at all, but added to Broad Sanctuary for the embellishment of London's most famous site. Governments, however, are never keen on non-political improvements, and so a commercial bidder must be awaited who will build. His letter appears elsewhere.

### ANNUAL MEETINGS IN THE PROVINCES.

The managers of the voluntary hospitals in the large provincial centres have for years shown the London voluntary hospitals how to make the annual meeting each year not only an asset but an awakener. Every effort is made to attract a large and representative audience, and to secure the attendance of many of the principal governors. It has long been regarded as a distinction to be selected as a member of the General Committee of one of the great voluntary hospitals in the provinces, and an attendance at the annual meeting in centres like Leicester and Birmingham, Newcastle, Liverpool, and Coventry, to mention only a few cities taken at random, is regarded as a privilege and duty not to be foregone. The Coventry Hospital has long had the good fortune to secure the services of one of the leading and most influential business residents as its chairman for two consecutive years at least. Thus Mr. A. Herbert, the former chairman, did yeoman service by securing the reconstruction of the hospital in many departments. He was succeeded by Mr. E. M. Iliffe, who has spared neither time nor influence in doing service for the hospital since he accepted office two years ago. The annual meeting on the 30th ult. will long be memorable from the large and representative number of governors who attended. Its proceedings had a special interest this year owing to the presence of Lord Northcliffe, a former resident in Coventry for health reasons, where he was associated in business with Mr. Iliffe's father. The material portions of Lord Northcliffe's interesting speech we published last week (see p. 394). We commend Lord Northcliffe's gift of £250 at the annual meeting to the governors of every voluntary hospital throughout London. We hope each of them may be specially invited to attend the annual meeting of his hospital during the present year. If this course be taken by the managers in 1914, and the annual meeting of every London hospital is provided with some special attraction calculated to enthuse its supporters, funds in goodly measure can be won for its treasury.

### MEDICAL EXCUSES.

HARD-WORKED practitioners have been known to offer varied excuses for non-attendance at emergency night calls, but one of the most curious was that offered last week by a medical man who refused to attend an accident case on the ground that it was one for the police surgeon. The unfortunate person seeking assistance thereupon turned to the police station, where the divisional police surgeon advised him to go to a third doctor. This last also refused, on the ground that the caller was not one of his patients. In reply to a question from the Coroner at the inquest the man thus turned away three times said that he had a panel doctor but fancied that he had no claim upon him. The Coroner then explained that a police surgeon's duties were to attend the force and cases sent in by its members. While regretting their behaviour, neither Coroner nor jury expressed any disapproval of the medical men's refusal to attend.

### LAMBETH GUARDIANS AND ACCOMMODATION FOR BABIES.

THE Lambeth Board of Guardians at their last meeting considered a proposal to remove the sick children in their infirmary at Brook Street to the children's infirmary at Norwood, and to transfer the weaned infants in Renfrew Road Workhouse to an annexe to the Home for Aged Poor at Norwood. Opponents urged that it was inhuman to remove children five miles away from their mothers. The new Order of the Local Government Board distinctly lays down that a mother shall have daily access to her infant, but the spirit of the Order will be violated if it is only to refer to mothers and children housed in the same building. The Rev. Walter Hobbs severely condemned the suggestion, which he characterised as inhuman. The Board agreed to the proposed alteration, but before it can be carried into effect a number of previous resolutions will have to be rescinded.

### DEATH OF A WELL-KNOWN LONDON GUARDIAN.

THE death of Alderman George Howlett, over thirty years a member of the Lambeth Board of Guardians, has removed from the front ranks of Poor-Law workers in South London one of the most prominent figures. From 1891 to 1907 he was chairman of the Lambeth Board, and during his *régime* many notable and far-reaching reforms were carried out. He had the honour of opening the buildings in Brook Street, Lambeth, now set apart for a nurses' home, an institution which will be long associated with his name. His personal history is curious, for he began life by hawking sacks of coke, which he carried on his own back to his customers; but by arduous efforts he lived to become one of the wealthiest men connected with Poor-Law work in London. Alderman Howlett, however, never assumed any "airs," but was to the last a thorough gentleman, plain and outspoken in acts and speech. He was a firm supporter and the friend of the many medical men associated with Poor-Law work in the parish, and by them will be greatly missed.

### FORTUNE FOR THE CANCER HOSPITAL.

HARD upon the Ansell bequests follows the announcement of the testamentary disposition of the fortune left by the late Mr. Thomas Cuvelje, formerly a stockbroker. The amount is sworn at over £110,000, of which the Surgical Aid Society and the Putney Hospital for Incurables get £5,000 apiece. There are other bequests stated to aggregate about £4,000, and the whole of the residue goes to the Cancer Hospital, Fulham Road. This will be about £96,000, less duty, which will, of course, be considerable; a truly magnificent legacy for a single institution. The Cancer Hospital is proverbially lucky in the matter of bequests; its aims and methods appeal very strongly to certain types of wealthy philanthropists. Of late years the governors have pursued a bold policy of expansion, and have expended large sums on research, radium and electro-therapy, and buildings. The result was that for 1912 the expenditure exceeded the ordinary income by £9,500; fortunately there were legacies totalling over £12,000 to make good the deficiency. This new windfall will provide, when invested, a very useful addition to the income of the charity; and justifies the managers in their energetic policy.

### UNNECESSARY INQUESTS AND THE WAY OUT.

A CORONER's jury manifested unusual public spirit at Walthamstow last week, when at an inquest on a man aged seventy-nine, whose death was medically certified to be due to senile dementia, they unanimously agreed that the cause of death being so clear an inquest was unnecessary. Dr. A. Ambrose, the Coroner, drew up a resolution, to be forwarded to the Home Office, to the effect that Coroners should be allowed more discretion to dispense with formal inquests when satisfied that the deceased has received all possible attention. Dr. Ambrose himself remarked that inquests of this kind were a waste of time and money, and instanced an experience of his own on the same day when being present at an inquest on a man aged ninety-one, the cause of whose death was obvious. He knew that a general desire existed for Parliament to pass an Act dealing with the matter. Legislation invariably follows the action and reaction of the human conscience in matters of the kind, and too much latitude is often followed by too much red tape. Before giving discretion to Coroners on a matter of such importance, a first step is to make their medical qualification compulsory.

### NON-PANEL DOCTOR'S SUCCESSFUL PROTEST.

Dr. Frederick Milnes Blumer, medical officer of health for the Borough of Stafford, was summoned last week by the Insurance Commissioners for failing to pay a contribution under Part I. of the Insurance Act in respect of his chauffeur, whose evidence showed that the doctor had refused to stamp his card on the local Insurance Committee declining to allow the witness to make his own arrangements for medical benefit. Dr. Blumer, a non-panel doctor, pleaded that the Commissioners' action in refusing to allow the local Committee to grant certain medical benefits under section 15 of the Act was *ultra vires*, and thus justified him in holding back his contributions until the refusal was cancelled or pronounced valid by the courts. The solicitor for the Commissioners admitted that permission to contract out was refused in this case, as Form 43, on the keeping of records, was not completed. Dr. Blumer stated that no medical man could retain his self-respect who signed that form. The Bench imposed a nominal penalty of one shilling and costs, which is a virtual victory for the energy displayed by this protest. That it should have come from a non-panel doctor is significant and of special interest to those who are enabled by our columns to follow the non-panel movement week by week.

### HOSPITAL CONTRIBUTIONS FROM APPROVED SOCIETIES.

A meeting was recently held at Edinburgh at which a recommendation was agreed to by the managers of the Royal Infirmary, delegates from the Trades' Council, and other bodies, that approved societies if possible should contribute to the infirmary from their surplus funds. Last week a meeting of the Trades' Council discussed it further, in the light of a recommendation from the delegates asking the Council to ratify the recommendation. Opinion was sharp and divided, Mr. Allan moving an amendment to the effect that it be not received favourably, on the ground that the proposal was simply meant to diddle the working-classes out of their benefits. Any surplus that the approved societies had should, he argued, be given back to their members. Mr. Eunsun stated that it was a wrong attitude to recommend approved societies not to give to the infirmary. There was, he thought, no difference in a working-man contributing as an individual and giving from the surplus fund of his approved society. The amendment was lost by 27 to 15, which presumably means that approved societies will be asked to contribute. There is no doubt that the gap which voluntary hospitals are filling in the Insurance Act by gratuitous provision of institutional treatment is one for which some cash return will have to be made to them sooner or later. The hospitals cannot dictate to any party, and need not wish to do so, since their work is so imperative to the success of the Insurance Act that neither societies nor the Government could afford to endanger their existence. The infirmary's managers have already urged their claim not only on the approved societies but also on the Burgh Insurance Committee.

### A PROJECTED PRIVATE HOSPITAL.

One of the most successful nursing homes in the West End of London is that in Henrietta Street which is managed by Dr. Bradford, who was at one time resident medical officer at St. Thomas' Home. In the *Lancet* this gentleman advocates the building of a large central private hospital for paying patients in London, to contain 150 to 200 beds. He holds that such a hospital could be run on lines which would permit of all excess profits, over a certain moderate percentage on the capital sunk, being devoted to relieving the fees of those who cannot afford normal rates. In a word, Dr. Bradford proposes a new Fitzroy House on a much larger scale; for there is no detail of his scheme, as at present disclosed, which has not been anticipated in the thirty-year-old institution at Fitzroy Square. After the object-lesson provided by the striking success of that private hospital it is astonishing that it should have remained unimitated so long. If the moment is ripe, as Dr. Bradford believes, for the erection of a private hospital on the ambitious scale he mentions, the lessons of experience will doubtless be kept in mind; and as a tried administrator of a home established in adapted premises, his requirements and the way in which his architect carries them out in a new building will be of great interest to institutional managers.

### THE POLICE AND THE HOSPITALS.

There has always been a good feeling between the Metropolitan Police and the hospitals—partly, no doubt, this is due to the assistance that the one can render to the other. The men of the police and the hospital staff must always be in close association. It is therefore with much gratification that a circular-letter has just been received by the hospitals of London from the Commissioner of Police, in which he intimates that, with the approval of the Home Secretary, he proposes to pay approximately a guinea a week for every policeman admitted as an in-patient. Sir Edward Henry, in his communication, goes out of his way to pay a graceful compliment to the hospitals. While recognising the services rendered in the past, he informs the hospitals that he is aware that the payment he now suggests does not represent the value of the medical and surgical treatment afforded, but he begs the Boards of Governors to accept the money as a voluntary contribution in recognition of his appreciation. There are other Government and municipal authorities which might well take a lead from the Commissioner of Police. The Post Office sends a large number of patients annually to the wards of the London hospitals. Telegraph and telephone men are particularly prone to accident, and it is with the greatest difficulty that the smallest recognition can be got from St. Martin's le Grand. Employees under the London County Council, the

Borough Councils, the Water Board all occupy a large number of beds. It will be found that a very small fraction of the cost of maintaining and treating these patients comes from the departments concerned.

### UNREST IN THE ASYLUMS BOARD AMBULANCE STAFF.

The motor-drivers and mechanics employed on the ambulance staff of the Metropolitan Asylums Board are endeavouring through the Municipal Employees Association to secure redress from the long hours and low salaries of which they complain. The Board's reply to the Association was that the ambulance staff should submit their demands through their station superintendents. The men appear unwilling to do this, but they have a capital opportunity now for urging their case with every legitimate emphasis, since the first meeting of the Board after the Christmas recess is to consider definite proposals for changes in the conditions of the ambulance service.

### COTTAGE HOSPITAL "AT HOMES."

A committeeman of the Acton Cottage Hospital suggests that greater support would be gained if the hospital were thrown open to the public, say for "at homes" once a month in winter, and twice a month in summer. If there were no serious cases in treatment, a local band might be invited to play in the grounds. A few cups of tea, he said, would not cost much, and such events would increase interest in their local hospital. The hospital possesses fine grounds, and the council are to consider the proposal. At the same meeting the treasurer of the hospital (Mr. Mark Pitt) reported that when all accounts had been paid there was a balance on the year of £120, whereupon Dr. Thornton remarked that such a result had not been attained for several years past. Functions connected with hospital developments are, of course, capital ways of stimulating interest, but hospital "at homes," at which the expenditure does not mark any new event, seem to us a questionable policy.

### ATTRACTIONS FOR RESIDENT MEDICAL OFFICERS.

The scarcity of resident medical officers for institutions is driving managers to all sorts of shifts for increasing the attractiveness of such posts. Already rates of pay have had to be raised in many cases; and other devices are receiving serious consideration which a few years ago would have been unheard of. Thus at the Swansea Hospital there is a small patch of grass which is dedicated to the house officers for use as a tennis court. This is, of course, only available for their recreation during the lawn-tennis season: roughly, May to September. So it has been proposed to lay down a gravel or asphalt court instead, which can be played on all the year round. The suggestion was supported on the ground that Swansea is an unattractive town to young men, and hence it is important to make the amenities of hospital life there as agreeable and inviting as possible. This statement about Swansea was not allowed to pass unanswered, and the local press is asking whether or not it is true. At all events, the question of altering the tennis court has not been definitely decided as yet, but is referred back to the house committee. Incidentally, a brick-dust court would probably be more suitable than either gravel or asphalt.

### CHELSEA HOSPITAL AND THE PALLADIUM.

The King and Queen hope to attend a matinée at the Palladium, on March 17, in aid of the rebuilding fund of Chelsea Hospital for Women. About the same date, all being well, Lord Cadogan's site for the new hospital should be available for building purposes, and, if so, the new institution will be completed within two years' time. The Coliseum performance in aid of Charing Cross and the French hospitals last year was so successful that the Chelsea Hospital should reap a large sum in March next towards the £18,000 still required for the rebuilding scheme.

### THE INSURANCE ACT AND THE DRUG BILLS.

In a number of districts—it is not as yet known precisely how many—the amount allotted for the payment of drugs for insured persons is not sufficient to pay the chemists' bills for the first year in full. The districts where the funds are likely to be deficient are, for the most part, in the North of England, but there are districts in other parts of the country where the Insurance Committees are faced by a similar problem. The one shilling and sixpence per head has been absorbed in the places indicated, as well as every penny of the floating sixpence, and still there is a deficiency which, we are sure, amounts to at least 30 per cent., but in other cases to much less. It is not definitely known whether the chemists will have to discount their bills, but, if so, the Insurance Act is not likely to gain in popularity among them. In many districts, on the other hand, the funds are enough to meet the bills in full and to pay, at any rate, a portion of the floating sixpence to the doctors.

### THIS WEEK'S DRUG MARKET.

The process of stock-taking is now completed and there is some improvement in business, although the market cannot correctly be described as very active even yet. The outlook, however, is hopeful, and there are more inquiries which may lead to business in due course. Of price changes of importance there have not been many during the week. Opium is practically unchanged, and it is thought that any alteration in price would probably be in an upward direction; the future of this market, however, is uncertain. A good business has been done in quinine. Cod-liver oil is still very dull. Menthol is quiet and practically unaltered in price. Belladonna root is dearer. Essence of lemon and oil of bergamot tend towards lower prices. Orris root is dearer. At the recent public sale of drugs in Mincing Lane the demand was quiet and few price changes of importance were recorded. Cape aloes sold at rates a trifle below those of the previous auction. Cardamoms had a somewhat lower tendency. Senna sold at practically unchanged prices.

